# General Statement in Regard to the Forsyth Dental Infirmary and Its Purposes

**Published:** 1923-07

**Authors:** 


### GENERAL STATEMENT IN REGARD TO THE FORSYTH DENTAL INFIRMARY AND ITS PURPOSES

The Forsyth Dental Infirmary for Children was founded by John Hamilton and Thomas Alexander Forsyth in memory of their brothers, James Bennett and George Henry Forsyth, and was incorporated in 1910 by a special act to the Massachusetts Legislature.

In formulating a working policy for its development the trustees decided at the outset that, to accomplish the greatest good, the new institution must aim at something higher than the mere repair and extraction of carious teeth, the correction of oral deformities and the treatment of adenoids and tonsils. It was recognized that the prevention of disease was equally, if not more, important than its treatment, and it was further thought that the institution should be developed on so high a plane of technical perfection that it should stand in the same relation to the dental profession that the hospital bears to the medical profession.

The objects of the institution as outlined in the previous reports relative to the education of the public, remedying existing conditions and establishing a higher standard of dental asepsis, are being accomplished with very gratifying results.

Without going into these subjects at tedious length, it was believed that, conducted upon the plan indicated, the institution would be of inestimable hygienic value to the rising generation of the children of Boston, and its vicinity; that it would instruct them, not only in oral hygiene but in general hygiene as well; that it would improve their nutrition and consequently their physical and mental growth; that it would lessen their liability to contract contagious and other diseases, and would place them in a better position to resist the same if contracted. And they further believed that the value of the institution to the dental profession would be great, not only from its research department, but also from its clinical branches. Unlike other practitioners of surgery, dentists have hitherto had little opportunity to observe the methods and practice of their colleagues, a dentist's practice being, for the most part, conducted in his office and by himself alone. An institution like the Forsyth Dental Infirmary, it was believed, would tend to counteract this narrowing tendency, and would contribute to establish a closer affiliation among members of the profession. It would furnish for dental practitioners an opportunity to observe a larger and more varied amount of clinical material; to view the work of others and to inspect new apparatus and appliances. Surgeons and physicians have long found it essential to success to set apart a portion of their time for free work in the hospitals; this institution should afford a similar essential opportunity to the dental surgeons.—From *Eighth Annual Report.*

